# A Longitudinal Computed Tomography Imaging in the Diagnosis of Gallbladder Cancer

**DOI:** 10.1155/2015/254156

**Published:** 2015-05-03

**Authors:** Atsuko Iwama, Shintaro Yamazaki, Yusuke Mitsuka, Nao Yoshida, Masamichi Moriguchi, Tokio Higaki, Tadatoshi Takayama

**Affiliations:** Department of Digestive Surgery, Nihon University School of Medicine, 30-1 Ohyaguchi Kami-machi, Itabashi-ku, Tokyo 173-8610, Japan

## Abstract

*Background/Aim*. To assess whether the diagnostic power of longitudinal multiplanar reformat (MPR) images is superior to that of conventional horizontal images for gallbladder cancer (GBC).* Methods.* Between 2006 and 2010, a total of 54 consecutive patients with preoperatively diagnosed gallbladder neoplasms located in gallbladder bed were analyzed. These patients underwent cholecystectomy with resection of the adjacent liver parenchyma. The patients were divided into the GBC group (*n* = 30) and the benign group (*n* = 24). MPR images obtained by preoperative multidetector row CT (MDCT) were assessed.* Results.* Mucosal line was more significantly disrupted in GBC group than that in benign group (93% [28/30 patients] versus 13% [3/24], *p* < 0.001). Maximum (9.3 [4.2–24.8] versus 7.0 mm [2.4–22.6], *p* = 0.29) and minimum (1.2 [1.0–2.4] versus 1.3 mm [1.0–2.6], *p* = 0.23) wall thicknesses on a single MPR plane did not differ significantly; however, the wall thickness ratio (max/min) differed significantly (6.8 [1.92–14.0] versus 5.83 [2.3–8.69], *p* = 0.04). Partial liver enhancement adjacent to tumor on longitudinal images was more common in GBC (40.0% [12/30 patients] versus 12.5% [3/24], *p* = 0.03). Mucosal line disruption was the most reliable independent predictor of diagnosis (odds ratio, 8.5; 95% CI, 5.99–28.1, *p* < 0.001).* Conclusion.* Longitudinal MPR images are more useful than horizontal images for the diagnosis of GBC.

## 1. Introduction

Despite recent advances in chemotherapy, outcomes of advanced gallbladder cancer (GBC) remain extremely poor as compared with those of other abdominal cancers. The 5-year survival rate of patients with advanced GBC is below 10% [1, 2]. The no residual of cancer has been shown to be the only factor contributing to outcomes, with a 5-year survival rate of 51% to 62% in such patients [3, 4]. However, R0 resection achieved in only 10% to 30% in advanced GBC [1, 3, 5].

The accuracy of preoperative diagnosis on conventional horizontal computed tomographic (CT) imaging alone has ranged from 52.6% to 71.7% [5–8]. Recently, the diagnostic accuracy of multiplanar reformatted (MPR) images obtained by multidetector row computed tomography (MDCT) was confirmed to be high in many abdominal organs [9–13]. The MPR imaging can provide optimal planes enabling evaluation of multiple aspects of lesions, with no loss of resolution. Thus, MPR imaging may have contributed to the preoperative diagnosis of GBC [5, 6].

The longitudinal image provides more detailed information for each layer of the organs than conventional horizontal CT [9–13]. Therefore, this imaging modality may have the ability to detect early GBC, such as T1 and T2 stages. Moreover, the tumor location, especially in the gallbladder bed, was found to be one of the risk factors for direct liver invasion and metastasis through the invasion of the venous plexus of the gallbladder bed [7, 8, 14]. The longitudinal image of the gallbladder can visualize the venous plexus as a layer.

Nonetheless, the evidence supporting the diagnostic value of a MPR image for GBC remains limited. The aim of this study is to assess the diagnostic value of early GBC and to assess the optimal procedure for GBC located in the gallbladder bed using longitudinal MPR imaging (along the major axis of the gallbladder).

## 2. Patients and Methods

### 2.1. Patients

The target population of this study was patients diagnosed with T1 and T2 stage GBC according to the preoperative conventional imaging. The location of the tumor was limited to the side of the gallbladder bed. The preoperative diagnostic workup included at least three different imaging modalities, selected from among abdominal ultrasonography, MDCT, magnetic resonance imaging (MRI), and endoscopic ultrasonography. This lesion was confirmed at least by two different preoperative diagnostic images. Four different estimators (two radiologists and two hepatobiliary surgeons [Atsuko Iwama and Shintaro Yamazaki]) working independently preoperatively assessed all conventional horizontal images and longitudinal MPR images.

The patients who refused this treatment strategy, had tumors located in the opposite side of the gallbladder bed, had marked liver invasion (T3 stage or worse), or had the presence of intrahepatic metastasis according to the preoperative imaging were excluded.

### 2.2. Surgical Procedure

At operation, intraoperative ultrasonography (IOUS) was performed in all cases. Concomitant liver resection was performed only when an obvious tumor was present at IOUS. Adequate surgical margins were confirmed by IOUS in cases that underwent liver resection. If a tumor was not confirmed by IOUS, cholecystectomy with Laennec's capsule (fibrous membrane around gallbladder) resection with the venous plexus of the gallbladder was performed. An intraoperative pathological evaluation using frozen sections was performed in all cases. When the malignant lesion was confirmed to be a lymph node or cystic duct, radical lymph node dissection with resection of the extra bile duct was performed.

All liver transections were performed by the clamp-crushing technique during intermittent clamping of the hepatoduodenal pedicle (Pringle's maneuver) for 15 min, followed by release for 5 min. During liver transection, the distance of the surgical margin from the liver tumor was checked by intraoperative ultrasonography to ascertain whether the margin was adequate. The details of the operative procedure have been described previously [15].

### 2.3. Protocol for Longitudinal MPR Imaging

Three-phase contrast-enhanced dynamic CT scans (unenhanced and hepatic arterial, portal venous, and liver parenchymal phases) were performed with a 16- or 64-detector row scanner (Aquellion 16/64; Toshiba Medical Systems, Tokyo, Japan). The total dose of nonionic iodinated contrast medium was 600 mgI/kg body weight of iomeprol (350 mgI/mL) or iohexol (300 mgI/mL), administered over the course of 30 seconds using an automatic injector (Type CD or Dual Shot GX; Nemoto Kyorindo, Tokyo, Japan) attached to a 20-gauge high-pressure intravenous (IV) catheter. Scanning was performed using a 15.0- or 53.0-helical pitch, a table feed speed of 0.75 or 0.5 mm per rotation, a slice thickness of 0.5 mm, 120 kV, and a Volume EC system (7.5 SD; Toshiba, Tokyo, Japan). After initiation of the contrast injection, scanning was performed in the arterial phase (37 seconds), in the portal venous phase (60 seconds), and in the delayed phase (150 seconds). Portal-phase images of row data were transferred to a workstation (System 1000; Ziosoft Inc., Tokyo, Japan), and MDCT images were reconstructed to coronal longitudinal images of the gallbladder with a section thickness of 1.00 to 2.00 mm without a gap by the MPR method. This protocol was approved by Ethics Committee of the Department of Digestive Surgery, Nihon University School of Medicine.

### 2.4. Assessment

**Figure 1:**
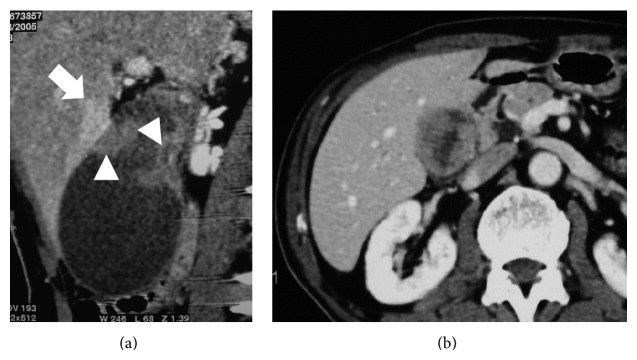
Longitudinal image of gallbladder cancer. Irregular mucosal enhancement with disruption of the enhanced mucosal line was visualized (arrowheads). Focal wall thickening and partial liver enhancement adjacent to the tumor were evident on longitudinal MPR imaging (arrow) (a). These phenomena were difficult to detect on horizontal images (b).

**Figure 2:**
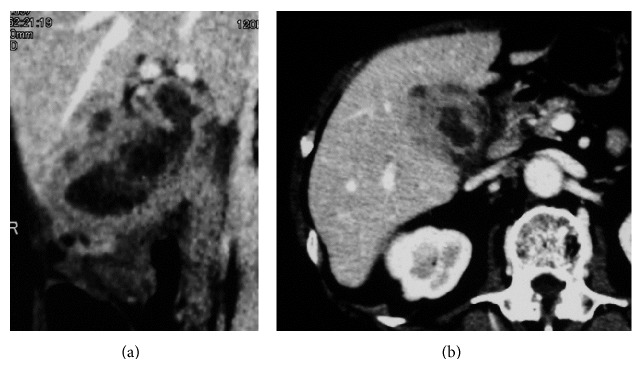
Longitudinal image of chronic cholecystitis. A case of cholecystitis mimicking gallbladder cancer. Uniform mucosal enhancement was observed. There was diffuse wall thickening with no parenchymal enhancement in the liver (a). The border between the gallbladder and liver parenchyma was unclear on horizontal images (b).

Images obtained by MDCT were reconstructed to longitudinal MPR images, allowing visualization along with the major axis of the gallbladder (Figures 1 and 2). The following findings of the gallbladder were assessed on longitudinal MPR images: calcification (gallbladder stones, gallbladder wall stones, biliary sludge, and common bile duct stones), the presence of hypodense bands between the gallbladder and liver parenchyma, mucosal line disruption, partial liver bed enhancement adjacent to tumor, the wall thicknesses, and the wall thickness ratio (maxim/minimum).

The relation between partial liver bed enhancement and histopathological invasion to the liver was examined on longitudinal images. Partial enhancement of liver parenchyma was defined as greater enhancement in liver parenchyma adjacent to tumor during the portal phase, with stagnation of enhancement during the liver parenchymal phase.

### 2.5. Statistical Analysis

Data are expressed as medians and ranges or as absolute values and percentages. Student's *t*-test, the *χ* ^2^ test, and Fisher's exact test were used for univariate analysis as required. Multivariate analysis was performed using logistic regression. Odds ratios with 95% confidence intervals derived from logistic regression were calculated. *p* values of <0.05 were considered to indicate statistical significance. All analyses were performed using a statistical software package (JMP version 8.0, SAS Institute Inc., Cary, NC, USA).

## 3. Results

### 3.1. Patients' Characteristics

**Figure 3:**
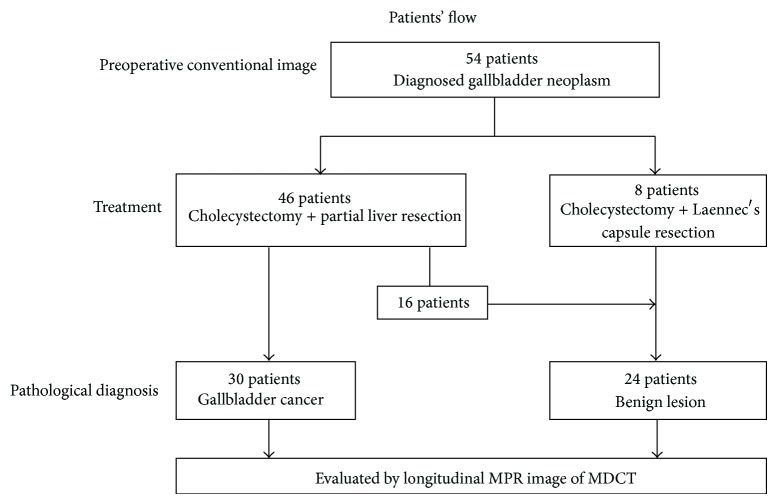


From July 2006 through August 2010, a total of 166 consecutive patients with gallbladder diseases were analyzed. 103 patients were preoperatively excluded by exclusion criteria in this study. Nine other patients were excluded because they obviously had a diagnosis of GBC. Ultimately, 54 patients were diagnosed with gallbladder neoplasms according to the preoperative conventional imaging and planned to undergo cholecystectomy with partial liver resection (Figure 3). Eight patients underwent cholecystectomy with Laennec's capsule (fibrous membrane around gallbladder) resection because there were no definitive malignant lesions according to IOUS. Forty-six patients underwent cholecystectomy with partial liver resection. Thirty patients were pathologically diagnosed with GBC (the gallbladder cancer group). The remaining 24 patients were designated as the benign group.

The serum alanine aminotransferase level (31 IU/L [6–1044] versus 18 [6–162], *p* = 0.03) and the *γ*-glutamyl transpeptidase level (99 IU/L [33–1009] versus 65 [13–584], *p* < 0.001) were significantly higher in the GBC group than in the benign group (Table 1). The carcinoembryonic antigen level was significantly higher in the GBC group than in the benign group (3.6 ng/mL [0.8–254.6] versus 1.9 [0.3–5.1], *p* = 0.01), whereas the CA 19-9 antigen level did not differ significantly (*p* = 0.48).

### 3.2. Longitudinal Image Analysis

On longitudinal MPR imaging, there were no significant differences between the GBC group and benign group in the presence of gallbladder stones, wall stones, biliary sludge, common bile duct stones, or hypodense bands between the gallbladder wall and liver bed (Table 2). Longitudinal MPR images revealed a disrupted mucosal line in 28 (93.3%) of the 30 patients with GBC, as compared with only 3 (12.5%) of the 24 patients with benign tumors (*p* < 0.001).

The maximum (9.3 mm [4.2–24.8] versus 7.0 [2.4–22.6], *p* = 0.29) and minimum (1.2 mm [1.0–2.4] versus 1.3 [1.0–2.6], *p* = 0.23) wall thicknesses on a longitudinal single plane did not differ between two groups, whereas the wall thickness ratio (maximum/minimum) on a longitudinal plane was significantly greater in the GBC group (6.8 [2.7–20.7] versus 5.8 [2.3–8.7], *p* = 0.04). The presence of partial liver parenchymal enhancement was more specific in GBC compared to that of benign group [10 patients (36.7%) versus 2 (12.5%), *p* = 0.03].

### 3.3. Association between Partial Liver Enhancement and Pathological Depth of Cancer

Thirty patients were pathologically diagnosed with GBC. Eleven patients had T1 tumors (T1a, 5 patients, and T1b, 6 patients), 10 patients had T2 tumors, and 9 patients had T3 tumors. Of the pathological T1 patients, none of the patient had partial liver enhancement of the liver parenchyma on the longitudinal images (Table 3). Of the pathological T2 patients, 3 out of 10 patients had partial liver enhancement of the liver parenchyma on the longitudinal images. In contrast, 8 of 9 patients (88.9%) of the pathological T3 patients had confirmed partial liver parenchymal enhancement on the longitudinal images.

### 3.4. Diagnostic Value for Gallbladder Cancer on Longitudinal Image

The diagnostic value of findings on longitudinal MPR images was evaluated by multivariate analysis. Mucosal line disruption was found to be an independent predictor of a diagnosis of GBC (odds ratio, 8.5; 95% CI, 5.99–228.1, *p* < 0.001) (Table 4).

### 3.5. Outcome of the Patients

There was no local recurrence and liver metastasis in the pathological T1a, T1b, and T2 patients. In contrast, 2 of the pathological T3 patients (*n* = 9) had liver metastasis within a year. However, no surgical site and peritoneal dissemination occurred.

## 4. Discussion

Our results demonstrated that longitudinal MPR image of the gallbladder has a diagnostic power of 91% for GBC. Mucosal line disruption was shown to be an independent predictor of diagnosis, with the highest odds ratio of 8.5 (95% CI, 5.99–28.1, *p* < 0.001).

MPR imaging can reconstruct multidirectional planes to target organs without loss of resolution. Previous studies have found that MPR imaging has high diagnostic accuracies for many abdominal diseases, including gastric cancer (85–89%), colon cancer (80–83%), and appendicitis (84–99%) [9–12]. The diagnostic accuracy (96%) of longitudinal MPR imaging of the extrahepatic bile ducts was shown to be higher than that of direct cholangiography (70%) in a recent series [13]. MPR imaging can depict the gradual transition from normal to abnormal tissue as estimated on the longest plane. In the diagnosis of GBC, each layer of the gallbladder is visualized along with the adjacent liver bed as long as possible. Thus, longitudinal MPR imaging appeared to provide a more objective plane than that obtained with horizontal imaging in the diagnosis of GBC.

It is well known that the two-stage operation for incidental GBC does not have a negative impact on the prognosis [7, 16, 17]. However, some studies mentioned about the risk of local site recurrence or had peritoneal dissemination in two stage operation [18, 19]. The tumor location, especially in the gallbladder bed, is one of the risk factors for direct liver invasion and metastasis through the invasion of the venous plexus of the gallbladder bed [7, 14]. Therefore, the optimal procedure for GBC, which is located in the gallbladder bed, is controversial. The longitudinal image of the gallbladder can visualize the venous plexus as a layer. Therefore, we analyzed patients diagnosed with GBC located in the gallbladder bed using preoperative imaging.

In present study, the longitudinal MPR images allow following 3 findings to be visualized in a single plane. (1) Mucosal disruption was the only independent predictor of GBC on multivariate analysis with the sensitivity 93.3% and the false negative rate of 6.7%. Mucosal spread and invasion are early events in carcinogenesis and may lead to mucosal disruption on longitudinal MPR image. (2) Diffuse thickening of the gallbladder wall suggests the presence of chronic cholecystitis because inflammation involves all layers of the wall [20]. In contrast, GBC develops focally without inflammation. The diagnostic accuracy of focal wall thickening in GBC was 77% [21, 22]. Longitudinal images of the gallbladder allow wall thickness to be evaluated in a single plane, thereby facilitating the distinction between diffuse and focal wall thickening. (3) Nine out of 30 pathological T3 patients had tumors underestimated as T2 according to the preoperative conventional images. Eight of these patients had partial liver parenchymal enhancement on the longitudinal image. Therefore, the partial liver enhancement may have the potential to predict liver invasion of GBC. In contrast, conventional horizontal images alone cannot accurately assess these variables.

According to our results, mucosal disruption in the diagnosis of GBC was mandatory. However, it is difficult to distinguish T2 tumors or T3 tumors. There was no local recurrence or liver metastasis in the pathological T1a, T1b, and T2 patients. The prognosis of GBC does not differ between the one- or two-step procedures in early GBC [7, 14, 16, 17]. Therefore, there is no need for liver resection in T1 or T2 tumors in the present study, and 16 patients in the benign group received more treatment than necessary. In contrast, surgical site recurrence and peritoneal dissemination did not occur in the pathological T3 patients who underwent partial liver resection. Therefore, we believe that the combined diagnosis of mucosal line disruption and partial liver enhancement demonstrated by longitudinal CT may contribute to the optimal treatment strategy for GBC. However, further studies using a larger sample size are necessary.

In conclusion, our results suggest that longitudinal MPR images oriented along a standard plane are useful for the diagnosis of GBC, with minimum false positive and negative results.

## Figures and Tables

**Table 1:** Patient characteristics.

	Gallbladder cancer	Gallbladder benign tumor	*p* value
	(*n* = 30)	(*n* = 24)	
Age	63 (42–77)	66 (19–82)	0.76
Gender (male)	15 (50%)	13 (54.2%)	0.76
Body mass index	22.9 (17.4–31.2)	22.7 (19.7–26.1)	0.62
White cell count (*μ*L)	6300 (4000–9100)	6850 (2500–16600)	0.21
Aspartate aminotransferase (IU/L)	31 (9–1688)	22 (11–174)	0.06
Alanine aminotransferase (IU/L)	30.5 (6–1044)	17.5 (6–162)	0.03
Lactic acid dehydrogenase (IU/L)	189 (109–1188)	168 (14–312)	0.13
Total bilirubin (mg/dL)	0.71 (0.34–13.96)	0.61 (0.12–3.27)	0.21
Alkaline phosphatase (IU/L)	222 (114–4399)	229 (115–1194)	0.93
*γ*-guanosine triphosphate (IU/L)	99 (33–1009)	65 (13–584)	<0.001
Cholinesterase (IU/L)	266 (93–458)	266 (166–450)	0.47
C-reactive protein (mg/dL)	0.5 (0.1–3.5)	0.5 (0.1–5.95)	0.88
Total cholesterol (mg/dL)	188 (144–324)	185 (133–266)	0.36
Prothrombin activity (%)	100 (79–100)	100 (73–100)	0.29
Carcinoembryonic antigen (ng/mL)	3.6 (0.8–254.6)	1.9 (0.3–5.1)	0.01
Carbohydrate antigen 19-9 (U/mL)	30.3 (0.1–9000)	40.0 (0.1–1056)	0.48

Data are expressed as medians with ranges.

**Table 2:** Longitudinal MPR image analysis.

	Gallbladder cancer	Gallbladder benign tumor	*p* value
	(*n* = 30)	(*n* = 24)	
Gallbladder stone	17 (56.7)	16 (66.7)	0.45
Gallbladder wall stone	10 (33.3)	13 (54.2)	0.12
Biliary sludge	10 (33.3)	14 (58.3)	0.07
Common bile duct stone	1 (3.0)	2 (8.3)	0.43
Hypodense band	9 (30.0)	13 (54.2)	0.07
Longitudinal mucosal line			
Continuous	2 (6.7)	21 (87.5)	<0.001
Disrupted	28 (93.3)	3 (12.5)	
Wall thickness			
Maximum^∗^ (mm)	9.3 (4.2–24.8)	7.0 (2.4–22.6)	0.29
Minimum^∗^ (mm)	1.2 (1.0–2.4)	1.3 (1.0–2.6)	0.23
Max/min ratio^∗^	6.8 (1.9–14.0)	5.8 (2.3–8.7)	0.04
Partial liver parenchymal enhancement	10 (36.7)	2 (12.5)	0.03

Data are expressed as numbers with percentages. ^∗^Median with range.

**Table 3:** Partial liver enhancement and pathological depth of cancer.

Pathological depth of invasion	T-stage	Number of patients	Partial liver enhancement	Proportion (%)
Mucosa	T1a	5	0	0
Mucosal plate	T1b	6	0	0
Subserosa	T2	10	3	30
Liver parenchymal invasion	>T3	9	9	88.9
Mild		1	1	100
Moderate		3	2	66.7
Massive		5	5	100

	Total	30	11	36.7

**Table 4:** Multivariate analysis of diagnosis on MPR image analysis.

	Multivariate analysis		
	Odds ratio	95% CI	*p* value
Longitudinal mucosal line disruption	8.5	5.99–28.1	<0.001
Maximum/minimum wall thickness ratio	3.5	0.62–25.1	0.16
Partial liver enhancement adjacent to tumor	2.1	0.3–17.7	0.45
Hypodense bands	1.1	0.41–3.3	0.61

## Abbreviations

MPR: Multiplanar reformat image
MDCT: Multidetector row CT.

## References

[1] Cubertafond P., Gainant A., Cucchiaro G. (1994). Surgical treatment of 724 carcinomas of the gallbladder: results of the French Surgical Association survey. *Annals of Surgery*.

[2] Kayahara M., Nagakawa T. (2007). Recent trends of gallbladder cancer in Japan: an analysis of 4770 patients. *Cancer*.

[3] Bartlett D. L., Fong Y., Fortner J. G., Brennan M. F., Blumgart L. H. (1996). Long-term results after resection for gallbladder cancer: implications for staging and management. *Annals of Surgery*.

[4] Chijiiwa K., Nakano K., Ueda J. (2001). Surgical treatment of patients with T2 gallbladder carcinoma invading the subserosal layer. *Journal of the American College of Surgeons*.

[5] Kalra N., Suri S., Gupta R. (2006). MDCT in the staging of gallbladder carcinoma. *American Journal of Roentgenology*.

[6] Kim S. J., Lee J. M., Lee J. Y. (2008). Accuracy of preoperative T-staging of gallbladder carcinoma using MDCT. *American Journal of Roentgenology*.

[7] Kokudo N., Makuuchi M., Natori T. (2003). Strategies for surgical treatment of gallbladder carcinoma based on information available before resection. *Archives of Surgery*.

[8] Ishikawa T., Horimi T., Shima Y. (2003). Evaluation of aggressive surgical treatment for advanced carcinoma of the gallbladder. *Journal of Hepato-Biliary-Pancreatic Surgery*.

[9] Shimizu K., Ito K., Matsunaga N., Shimizu A., Kawakami Y. (2005). Diagnosis of gastric cancer with MDCT using the water-filling method and multiplanar reconstruction: CT-histologic correlation. *American Journal of Roentgenology*.

[10] Chen C.-Y., Hsu J.-S., Wu D.-C. (2007). Gastric cancer: preoperative local staging with 3D multi-detector row CT—Correlation with surgical and histopathologic results. *Radiology*.

[11] Filippone A., Ambrosini R., Fuschi M., Marinelli T., Genovesi D., Bonomo L. (2004). Preoperative T and N staging of colorectal cancer: accuracy of contrast-enhanced multi-detector row CT colonography—initial experience. *Radiology*.

[12] Miki T., Ogata S., Uto M. (2005). Enhanced multidetector-row computed tomography (MDCT) in the diagnosis of acute appendicitis and its severity. *Radiation Medicine*.

[13] Akamatsu N., Sugawara Y., Osada H. (2009). Preoperative evaluation of the longitudinal spread of extrahepatic bile duct cancer using multidetector computed tomography. *Journal of Hepato-Biliary-Pancreatic Surgery*.

[14] Sugita M., Ryu M., Satake M. (2000). Intrahepatic inflow areas of the drainage vein of the gallbladder: analysis by angio-CT. *Surgery*.

[15] Yamazaki S., Takayama T., Kimura Y. (2011). Transfusion criteria for fresh frozen plasma in liver resection: a 3 + 3 cohort expansion study. *Archives of Surgery*.

[16] Wakai T., Shirai Y., Hatakeyama K. (2002). Radical second resection provides survival benefit for patients with T2 gallbladder carcinoma first discovered after laparoscopic cholecystectomy. *World Journal of Surgery*.

[17] Araida T., Higuchi R., Hamano M. (2009). Should the extrahepatic bile duct be resected or preserved in R0 radical surgery for advanced gallbladder carcinoma? Results of a Japanese Society of Biliary Surgery Survey: a multicenter study. *Surgery today*.

[18] Z'Graggen K., Birrer S., Maurer C. A., Wehrli H., Klaiber C., Baer H. U. (1998). Incidence of port site recurrence after laparoscopic cholecystectomy for preoperatively unsuspected gallbladder carcinoma. *Surgery*.

[19] Kim P. N., Ha H. K., Kim Y. H., Lee M.-G., Kim M. H., Auh Y. H. (1998). US findings of xanthogranulomatous cholecystitis. *Clinical Radiology*.

[20] Goshima S., Chang S., Wang J. H., Kanematsu M., Bae K. T., Federle M. P. (2010). Xanthogranulomatous cholecystitis: diagnostic performance of CT to differentiate from gallbladder cancer. *European Journal of Radiology*.

[21] Uchiyama K., Ozawa S., Ueno M. (2009). Xanthogranulomatous cholecystitis: the use of preoperative CT findings to differentiate it from gallbladder carcinoma. *Journal of Hepato-Biliary-Pancreatic Surgery*.

[22] Chun K. A., Ha H. K., Yu E. S. (1997). Xanthogranulomatous cholecystitis: CT features with emphasis on differentiation from gallbladder carcinoma. *Radiology*.

